# Empirical Evaluation of UNet for Segmentation of Applicable Surfaces for Seismic Sensor Installation

**DOI:** 10.3390/jimaging12010034

**Published:** 2026-01-08

**Authors:** Mikhail Uzdiaev, Marina Astapova, Andrey Ronzhin, Aleksandra Figurek

**Affiliations:** 1 St. Petersburg Federal Research Center of the Russian Academy of Sciences (SPC RAS), 39, 14th Line, St. Petersburg 19917, Russia; uzdyaev.m@iias.spb.su (M.U.); astapova.m@iias.spb.su (M.A.); ronzhin@iias.spb.su (A.R.); 2 School of Business, GNOSIS Mediterranean Institute for Management Science, University of Nicosia, Nicosia 1700, Cyprus

**Keywords:** multispectral data, Sentinel-2, automation of seismic sensor deployment, U-Net architecture, semantic segmentation, remote sensing

## Abstract

The deployment of wireless seismic nodal systems necessitates the efficient identification of optimal locations for sensor installation, considering factors such as ground stability and the absence of interference. Semantic segmentation of satellite imagery has advanced significantly, and its application to this specific task remains unexplored. This work presents a baseline empirical evaluation of the U-Net architecture for the semantic segmentation of surfaces applicable for seismic sensor installation. We utilize a novel dataset of Sentinel-2 multispectral images, specifically labeled for this purpose. The study investigates the impact of pretrained encoders (EfficientNetB2, Cross-Stage Partial Darknet53—CSPDarknet53, and Multi-Axis Vision Transformer—MAxViT), different combinations of Sentinel-2 spectral bands (Red, Green, Blue (RGB), RGB+Near Infrared (NIR), 10-bands with 10 and 20 m/pix spatial resolution, full 13-band), and a technique for improving small object segmentation by modifying the input convolutional layer stride. Experimental results demonstrate that the CSPDarknet53 encoder generally outperforms the others (IoU = 0.534, Precision = 0.716, Recall = 0.635). The combination of RGB and Near-Infrared bands (10 m/pixel resolution) yielded the most robust performance across most configurations. Reducing the input stride from 2 to 1 proved beneficial for segmenting small linear objects like roads. The findings establish a baseline for this novel task and provide practical insights for optimizing deep learning models in the context of automated seismic nodal network installation planning.

## 1. Introduction

In recent years, interest in wireless seismic nodal systems has grown significantly. These systems enable the rapid deployment of sensor networks across terrain without the need for cable laying [1,2,3,4]. A key challenge in deploying such autonomous seismic networks is selecting optimal locations for the modules. This selection must consider factors such as site accessibility, ground stability, and the absence of acoustic and seismic interference [5,6,7,8]. Despite significant progress in remote sensing and deep learning methods, the field of semantic segmentation has also advanced. These techniques are now widely used for classifying land cover, infrastructure, and natural objects [9,10]. However, research on the automated semantic segmentation of zones specifically suitable for installing seismic sensors is currently scarce. In contrast, most existing studies focus on general segmentation tasks. These tasks include landscape, road, water body, and urban environment segmentation [11,12,13,14]. Automating the search for applicable locations for seismic sensor installation using semantic segmentation methods could significantly enhance the efficiency and accuracy of deploying seismic networks. This is particularly relevant for remote or vast areas [15,16]. Consequently, the lack of specialized deep learning models and methodologies for the semantic segmentation of suitable zones underscores a clear research gap. It highlights the relevance and necessity for developing new approaches specifically aimed at finding optimal sites for seismic sensor placement.

It is important to emphasize that here the “suitability” of the terrain is not defined only as a standard class of land coverage, but as a functional criterion related to the practical conditions for the installation of seismic nodal sensors. This is not “another” landscape segmentation, but an attempt to translate satellite segmentation directly into an operational decision (where a sensor can and cannot be placed in real terrain).

Unlike the general tasks of semantic segmentation in remote sensing, the goal of this work is not land cover classification for cartography or urban mapping, but solving the specific operational problem of planning the field layout of seismic sensors. In this context, semantic segmentation is used as a tool for preliminary field assessment of surfaces that are functionally suitable or unsuitable for the installation of seismic nodal modules. Therefore, the classification criteria are not based exclusively on the spectral characteristics of the objects, but also on their practical suitability for seismic measurements, including the stability of the substrate, the presence of anthropogenic disturbances, and the accessibility of the terrain. This formulation of the task introduces additional complexity: the boundaries between classes are often not “purely spectral”, but also depend on the context (e.g., anthropogenic noise, access, stability of the substrate). Therefore, performance on rare and borderline classes is expected to be lower than in classic benchmark segmentation tasks, and the results must be interpreted as a realistic baseline, not as a “ceiling” of possible performance.

The preliminary reconnaissance of surfaces suitable for seismic sensor installation relies on a key step: the analysis of multispectral satellite imagery. This approach is highly promising due to the wide availability of such data, its high update frequency, and its diversity of spectral channels [17]. However, effectively training deep learning models requires labeled data. This data must account for the specific characteristics of the target surfaces from the perspective of seismic sensor installation. The challenges of forming a representative sample and creation developing automated labeling methods for such data were addressed in our previous work [5]. In the context of this paper, the emphasis is not on a “new architecture”, but on establishing a transparent and comparable experimental protocol on a dedicated dataset. This provides a reference point for future improvements (e.g., different losses, class balancing, additional models), with clearly defined terms of comparison.

This work is devoted to baseline empirical evaluation of the various UNet [18] architecture modifications on the created dataset to perform the semantic segmentation of zones that are optimal for seismic sensor installation. The main contributions of this work are the following. Formulation and empirical analysis of a specialized task of semantic segmentation aimed at the identification of surfaces suitable for the installation of seismic sensors, which represents a topic that is currently insufficiently represented in the literature in the field of remote sensing. Baseline evaluation of the widely used U-Net architecture for semantic segmentation on a dedicated multispectral Sentinel-2 dataset, annotated in the context of seismic sensor deployment. The study systematically analyzes the impact of: (a) different pre-trained encoders (EfficientNet, CSPDarkNet53, MAxViT), and (b) multiple combinations of Sentinel-2 spectral channels with different spatial resolutions. Empirical evaluation of a simple input layer modification strategy, based on changing the stride of the input convolutional layer from 2 to 1, with the aim of improving the segmentation of small and linear objects relevant for seismic installation, such as roads and debris. The contribution of this work is not reflected in the development of a new architecture of deep learning, but in the introduction and empirical analysis of the specialized task of semantic segmentation of surfaces suitable for the installation of seismic sensors. A key contribution is a dedicated labeled dataset based on multispectral Sentinel-2 imagery, created and manually verified in the context of seismic applications. Based on this dataset, the paper provides a systematic empirical benchmark of variants of the U-Net architecture with different pre-trained encoders, combinations of spectral channels, and changes in the input convolutional stride. Although the methods used are not new in themselves, their comparative evaluation in this specific context enables the establishment of a reference base (baseline) for future research and practical applications in the planning of seismic nodal networks. The novelty is, above all, in the domain-specific definition of “suitability” and its mapping into pixel-level segmentation on multispectral Sentinel-2 images, which differs from standard land-cover segmentation.

## 2. Related Work

Automated mapping using satellite data for the searching of applicable places for seismic sensor installation tasks addresses the logistical challenges of deploying wireless network nodes. In this section, we group the works according to what they solve: (i) sensor placement optimization, (ii) semantic segmentation of satellite images for object and infrastructure mapping, and (iii) techniques that improve the quality of input data or model generalization (synthetic data, multi-temporal fusion, super-resolution, and foundation models). This division is important because our problem is not the classic land-cover segmentation, but the segmentation of surfaces defined by functional suitability for seismic measurement. The study by [19] utilized a deep neural network (DNN) to optimize seismic sensor placement. This DNN was trained on the synthetic dataset of accelerograms from PEER NGA-West2 [20]. Works dealing with the optimization of seismic sensor placement often assume that the terrain conditions are known or generated (e.g., synthetic). In this sense, they solve “where to place sensors within the wave/reconstruction model”, but they do not solve the previous operational step: how to automatically extract from satellite data the zones that are generally suitable for physical installation (substrate stability, anthropogenic noise, and accessibility). A four-sensor configuration demonstrated the best performance, achieving a reconstruction error of 4.69%. Semantic segmentation methods for satellite imagery are advancing in cartography and land cover classification. These techniques enable the identification of natural hazard zones and anthropogenic features, optimizing the placement of sensors and monitoring system components. In the work of [21], a U-Net model [17], trained on the Semantic Segmentation of Aerial Imagery dataset [22], achieved an F1-score of 0.72 for extracting built-up areas in landslide zones. The work by [23] proposed a method for the automated mapping of linear disturbances, such as roads, pipelines, and seismic lines. The approach, based on VGGNet-16 [24] and U-Net, achieved F1-scores of 0.969 for the background class and 0.776 for roads. The generation of synthetic data is another active research area. The authors of [10] introduced a method for generating synthetic data for Sentinel-2 image segmentation. In their work, a DeepResUNet model [25] achieved an IoU of 90–100% for major land cover classes. Multi-temporal data fusion also shows significant promise. The approach in [26] used multiple Sentinel-2 revisits and a SWIN Transformer with Latent Fusion, achieving an IoU of 0.58 for segmenting electrical substations. Improving image resolution is crucial for detailed analysis. The authors of [27] presented a hybrid architecture called Sen4x for enhancing the resolution of Sentinel-2 imagery. Their model outperformed Swin2SR [28] and ESRGAN [29], achieving an accuracy of 0.746 and an mIoU of 0.516. Multimodal fusion is another trend. The work [30] proposed the LF-DLM model, which fuses aerial imagery with the Sentinel-2 time series. On the FLAIR #2 dataset [31,32], models and one U-TAE [33] achieved an mIoU of 64.52. The authors [34] developed SemESRGAN, a new method for super-resolution aerial image restoration. This technique incorporates semantic segmentation. Evaluations on six datasets showed that SemESRGAN achieves higher reconstruction quality. The improvement was confirmed by higher PSNR and SSIM scores. The method outperformed baseline models based on convolutional networks, visual transformers, and generative adversarial networks.

Transfer learning from foundation models is also being explored. The research work [35] introduced the TransLandSeg approach for landslide segmentation, leveraging the SAM model [36]. On the Bijie Landslide dataset [37], the model reached an MIoU of 88.10% and an F1-score of 93.41%. Approaches based on “foundation” models and transfer learning show the potential for faster adaptation to new domains. And in those works, the target class is typically a clearly defined visual category (e.g., landslides), while our target designation is a combination of visual indicators and practical seismic criteria. It is crucial to have a dedicated annotated dataset and a transparent evaluation protocol, so that future methods (including foundation models) can be fairly compared under the same conditions.

Numerous other studies have demonstrated high performance in land cover classification tasks using various models and data configurations, including SVM classifiers [38] achieving 98.05% overall accuracy, MRUNet for identifying flooded corn [39], MSNet for multispectral images [40], LinkNet-ResNet34 for land cover [12], and U-Net with combined spectral and index channels [41]. High-performance results have also been reported for applications like wildfire burn scar mapping [42], multispectral image segmentation with PSNet [43], water body segmentation [44], and land use classification with multimodal data [45]. Ref. [46] presented an approach for verifying oil spills on water surfaces using deep learning [2]. Their method uses a Siamese network with a lightweight VGG16 for classification. For segmentation, it employs the Poly-YOLOv3 [47] architecture. This strategy resulted in a classification accuracy of 0.91. It also yielded a segmentation accuracy of 0.97.

The field of satellite data semantic segmentation is rapidly evolving. It shows significant achievements in segmenting various land cover types, such as urban areas, roads, water bodies, and agricultural land. This progress is driven by architectures, including networks, transformers, and approaches, as well as training methods utilizing synthetic data. A specific research gap remains; the literature lacks studies dedicated to the specialized task of semantically segmenting zones suitable for installing seismic sensors. Existing models and datasets only partially overlap with the surface classes relevant to the mentioned task. These relevance criteria include soil quality, distance from noise sources, and terrain traversability and accessibility. While current datasets cover general land cover classes, practically no specifically labeled datasets exist for seismological purposes. Consequently, developing methods for the semantic segmentation of applicable surfaces for seismic sensor installation from satellite imagery remains a relevant challenge. This development is essential for the specific task of terrain reconnaissance aimed at deploying seismic sensors, particularly considering the specific characteristics of multispectral satellite data. The source code for this article is available in Supplementary Materials section.

## 3. Materials and Methods

### 3.1. Methodology of the Research

In this work, we perform a baseline study of a specific dataset that contains labeled multispectral satellite images with applicable and non-applicable surfaces for seismic sensor installation [5]. In this work, we consider a wide-spread UNet [18] architecture as a baseline model. We study the combinations of the following UNet parameters:

1. Type of pretrained encoder. We consider two convolutional neural networks (CNN) as encoders: EfficienNetB2 [48] and CSPDarknet53 [49], and one Vision Transformer (ViT) architecture: MAxViT [50].
2. The following spectral band combinations of Sentinel-2 multispectral bands: RGB (3 bands with 10 m/pix spatial resolution), RGB+NIR (4 bands with 10 m/pix spatial resolution), all bands with 10 and 20 m/pix spatial resolution (10 bands), and full spectrum of Sentinel-2 (13 bands with 10, 20, and 60 m/pix resolution).
3. Two values of the input convolutional layer strides (stride = 1 and stride = 2) in order to evaluate a technique for small object segmentation improvement, that will be thoroughly described in Section 3.6: “Improving small object segmentation”.

In this work, we obtained results for 24 combinations (3 encoders, 4 Sentinel-2 band combinations, and 2 values of the input convolutional stride). We analyze the following outcomes of the experiments:

1. The impact of a pretrained encoder used for feature extraction. In order to do this, we consider all possible band and stride combinations and search for the best encoder for each of the surface classes according to each metric.
2. The impact of different multispectral band combinations. In order to do this, we compare results of the corresponding classes for combinations of encoders and input the convolutional layer stride. First, we compare results with each encoder, then we summarize the results of the considered encoders.

An impact of the input layer convolutional stride. In order to do this, first, we consider each encoder separately and compare the results of each metric of each class between spectral band combinations and define the best stride value, then we define the best stride values between classes for each spectral band combination. Finally, we summarize the results of the considered encoders. In this research, all models were trained under identical experimental conditions to ensure a fair and comparable evaluation of different configurations. The input multispectral channels are prenormalized independently per channel, without applying additional complex augmentation techniques, in order to maintain a realistic relationship between the spectral characteristics of the surfaces and their seismic suitability. A multi-class cross-entropy loss function without class weighting was used for training. The spatial separation of the data is ensured, whereby no part of the same geographical area is simultaneously present in the training and test set. This approach prevents information leakage (data leakage) between sets and enables a more realistic assessment of model generalization to spatially independent areas, which is especially important in the context of planning seismic field campaigns.

The scientific depth of this review is in the systematic benchmark (24 configurations) with a strictly controlled training protocol and spatially independent evaluation, thus providing a reliable reference point (baseline) and eliminating methodological ambiguities that often make comparisons in the literature unfair or non-reproducible.

### 3.2. Dataset Description

**Figure 1:**
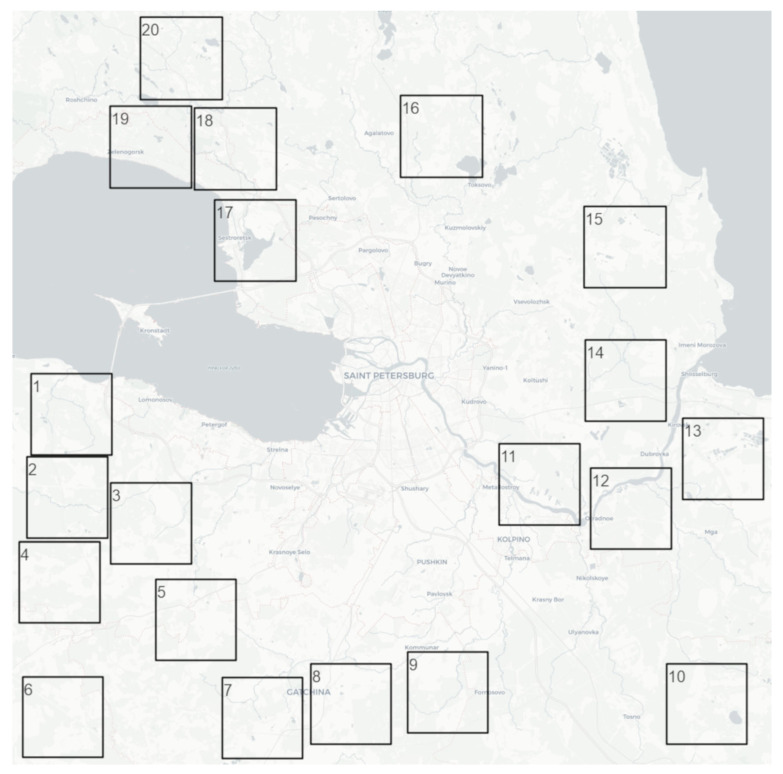
Geographical locations of the images in the dataset. 1–20 are the ordinal numbers of the squares in the dataset that are used in the partition into training and test split.

This paper presents the results of semantic segmentation on a dataset of surfaces suitable for seismic sensor installation [5]. The dataset consists of Sentinel-2 Level L2A multispectral satellite imagery. These images cover 20 geographical locations near Saint Petersburg. Each location covers an area of 10.5 × 10.5 km. This area corresponds to images of 1050 × 1050 pixels in size, with a spatial resolution of up to 10 m/pixel. Each image is represented by 13 Sentinel-2 spectral channels, which range from 443 to 2190 nm. The total covered geographical area is 2205 km^2^. The size of the obtained area covered by the images is determined by the limitations of Sentinel Hub API trial access https://www.sentinel-hub.com/develop/api/ (accessed on 19 December 2025). The location selection followed several criteria. First, the scenes must contain land cover classes relevant to the task of applicability for seismic module installation. Second, the dataset must include diverse surface types. Third, the images were selected from summer months to exclude seasonal distortions. Furthermore, the selected scenes are free of clouds. Finally, the dataset requires the presence of detailed OpenStreetMap (OSM) vector labeling https://www.openstreetmap.org (accessed on 19 December 2025). The locations of the satellite images are shown in Figure 1.

**Figure 2:**
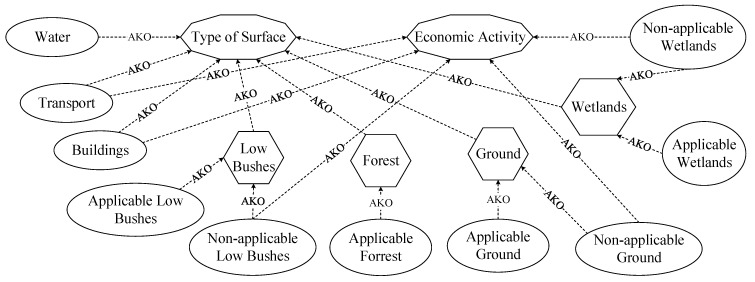
Relations between classes of applicable and non-applicable surfaces. AKO is “a kind of” or an inheritance relationship.

A class system of applicable surfaces for seismic sensor installation was developed for object classification. This system accounts for differences in surface types regarding their suitability for seismic sensor installation. The list of all considered surface classes is as follows: Transport (paved and unpaved roads, railways); Buildings (urban, rural, and industrial development); Water (rivers, lakes, seas, and artificial reservoirs); Wood (natural forest areas); Low bushes (shrub thickets and unused clearings, as well as similar areas with agricultural crops and pastures); Wetland (natural marshes and bogs, and peat production sites); and Ground (bare soils, sandy beaches, and similar territories with industrial exploitation). Figure 2 illustrates the relationships between applicable and non-applicable surface classes. The applicability of each surface type depends on its classification for seismic module installation. The suitability criterion was defined as the presence of economic activity on a given surface, which prevents the installation of seismic sensors.

The image labeling is based on OSM vector georeferenced data. These data were used for the initial labeling of surface classes. In order to align the OSM classes with the domain-specific surface classification system of applicable surfaces, a mapping procedure was applied [5]. Additionally, inspection of objects was performed by experts. The need for expert inspection arose from several issues. These issues include errors in community-based OSM labeling, missing labels, and conflicts arising from multiple classifications of the same object. The expert inspection procedure was carried out in the QGIS environment [51]. It consisted of the following operations: merging similar objects; correcting object classes; adding objects where they were missing in the original labeling; resolving object intersections; and removing irrelevant objects. The inspection was performed by comparing the OSM basemap with detailed satellite imagery, such as Google Earth Pro [52] and Retromap [53]. This expert verification procedure was applied to images acquired on 23 September 2023 [5]. The dataset statistics used in current research is presented in Table 1. In order to enlarge the number of training images, each of the 20 areas (Figure 1) are split into 96 × 96 multispectral images (2420 images in total).

The dataset was divided into training and testing subsets in a such way that 70% of squares (i.e., 14 that contain 1694 96 × 96 images) depicted in Figure 1 were chosen for the training set and 30% of squares (i.e., 6 that contain 726 96 × 96 images) were chosen for the test set. Namely, squares with numbers 3, 4, 9, 14, 17, 18 were utilized in the test set. In order to obtain the closest class distributions in the train and test subsets, we (1) obtained all the nk=n!/k! n−k! combinations of *k* = 6 test squares of a full set of *n* = 20 squares depicted in Figure 1; (2) iterate over all the obtained combinations, and compute the distributions of classes in train and test subsets; and (3) compute the Euclidean distance between vectors of the combinations of class distributions and a vector of the class distribution of the initial dataset, and choose the combination with the minimal distance. The class distributions of the train and the test subsets are presented in Table 1.

Class labels were initially generated based on OpenStreetMap (OSM) vector data. OSM is a community-labeled geospatial dataset that suffers from subjective errors of non-professional labelers. In order to overcome the initial subjective bias of the OSM community, the dataset was then manually corrected to accommodate specific seismic application criteria. This process also introduces a certain degree of subjectivity, especially in borderline cases where the boundaries between classes are not clearly defined. Manual corrections were made by domain experts with visual verification in a GIS environment and comparison with high-resolution satellite images. A formal analysis of the agreement between multiple annotators was not performed, which is a limitation of the dataset. However, a quality control (QA) procedure was applied that included multiple visual inspections, the correction of obvious errors, and the removal of conflicts between overlapping labels. Although the presence of mislabels cannot be completely ruled out, it is estimated that the gross mislabeling rate is limited and does not dominate the overall behavior of the model during training and evaluation.

The dataset used is characterized by a marked imbalance between classes. Certain classes, such as “Applicable Wood”, make up almost half of the total number of marked pixels, while others, such as “Applicable Ground”, are represented by less than 1% of the total area. This imbalance reflects the real structure of the considered geographical area, but has a significant impact on the behavior of the model during learning. Models trained on such data tend to favor dominant classes, which can lead to lower values of IoU and recall metrics for rare classes. For this reason, the results for poorly represented classes should be interpreted with special caution, and the average metrics should be viewed primarily as indicative of the overall behavior of the model, and not as equally representative of all classes.

### 3.3. UNet Encoder–Decoder Architecture

**Figure 3:**
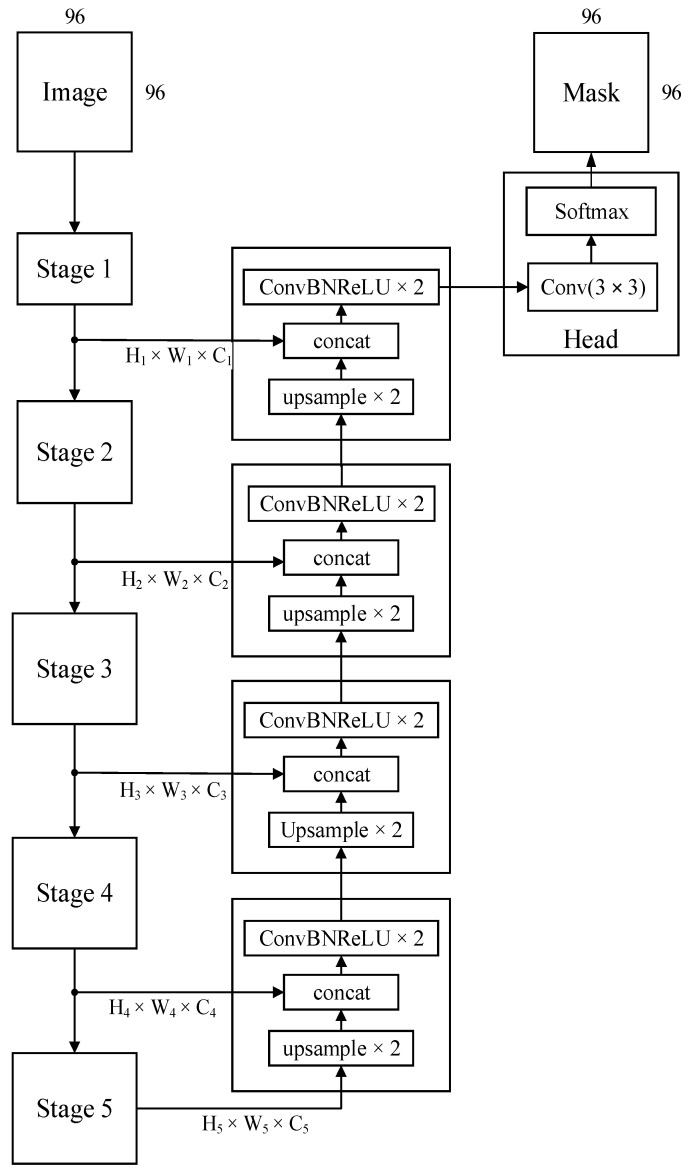
UNet architecture.

In this work, we utilized encoder–decoder UNet architecture with skip connections or shortcuts for area localization while leveraging contextual information on different levels of image processing [18] (Figure 3).

The network processes an input image with a spatial dimension of 96 × 96 pixels. The number of input channels depends on the various channel configurations. In this work, we consider the following input channel configurations: RGB (3 channels), RGB+NIR (4 channels), channels with 10 and 20 m/pix spatial resolution (10 channels), and all 13 Sentinel-2 multispectral channels.

The architecture can be divided into two primary pathways: an encoder for feature extraction and a decoder for segmentation map generation. The encoder consists of four sequential stages (Stage 1 to Stage 4). The architecture of each stage inherited from the architectures of pretrained CNN utilized for feature extraction. A downsampling operation follows each stage, halving the spatial dimensions and doubling the number of feature channels, as indicated by the changing dimensions from H_1_ × W_1_ × C_1_ to H_4_ × W_4_ × C_4_. This hierarchical downsampling allows the network to learn features at various scales, from fine-grained details to more abstract representations. The last stage of the encoder (H_5_ × W_5_ × C_5_) acts as the bottleneck of the network. This stage operates on the most compact feature representation, capturing high-level semantic information. The decoder mirrors the encoder and similarly comprises four stages. Each stage in the decoder begins with a 2× upsampling operation, which increases the spatial resolution of the feature maps. Crucially, the upsampled features are then concatenated with the corresponding feature maps from the encoder pathway via skip connections. These connections, denoted by “concat,” transfer high-resolution spatial information from the encoder to the decoder, enabling the network to recover fine-grained details lost during downsampling. Following each concatenation, two blocks of Convolution, Batch Normalization, and ReLU activation (ConvBNReLU) are applied to refine the combined features. The final (head) layer of the decoder produces a feature map with the same spatial resolution as the input image, followed by a 3 × 3 convolution to map the high-dimensional features to the desired number of output classes, resulting in a pixel-wise segmentation map. The output is the same size as the input. The number of classes is equal to 11.

In this work, we focus on study encoders and preprocessing parts, rather than on the study of various semantic segmentation architectures. UNet is a well-developed, relatively lightweight architecture that requires less computational resources than UNet++ [54] and transformer segmentation architectures [55,56]. Moreover, UNet has the ability of input layer adaptation for small object handling that will be described in Section 3.5 [54,55,56,57].

### 3.4. Description of Encoders

In this work, we follow the principle implemented in Segmentation Models Pytorch library [58]. This principle implies that layers of the pretrained image processing neural network are divided into five consecutive stages. Each stage consists of several convolutional layers. After every stage, the encoder reduces the spatial size by half, and usually increases the number of feature maps. The architecture and weight values of these layers are inherited from the original pretrained neural network architecture. The produced feature maps by each stage are fed into the corresponding layers of decoder architecture. The described principle makes it possible to utilize a wide set of pretrained image processing neural network architectures that may potentially improve segmentation results.

In this work, we consider EfficienNetB2 [48], CSPDarknet53 [49], and MAxViT [50] as image encoders. All the considered architectures are pretrained on the Imagenet 1K dataset [59] for a classification task of a large number of objects.

#### 3.4.1. EfficientNetB2

The main distinctive feature of EfficientNet is Mobile Inverted Bottleneck Convolutional Blocks. These blocks are a modification of the Residual Blocks [60] and Inverted Residual Blocks [61]. The input features are expanded to a given number of channels using 1 × 1 convolution. Then, these feature maps are fed into a Squeeze-and-Excitation (SE) module [62] that implements the channel attention mechanism. The channels of the features, processed by the SE module, are then reduced using 1 × 1 convolution. The output features are summated with the input features. The hyperparameters (such as channel number, layer number, block structure, etc.) of EfficientNet architectures are optimized using the Neural Architectural Search technique.

#### 3.4.2. CSPDarkNet53

CSP (Cross-Stage Partial) Networks [49] consist of CSP Blocks. Each CSP Block processes the input convolutional features using two branches. The first branch applies convolution to the input feature maps and produces feature maps with half of the number of initial channels. The second part also produces a half-channel number feature map that is fed into a sequence of Residual Blocks. The output features maps are formed by concatenation of the feature maps produced by the first branch and feature maps produced by the second branch.

#### 3.4.3. MAxViT

In this work, we also utilized Multi-Axis Vision Transformer (MAxViT) architecture. This architecture comprises three consecutive modules: an Inverted Residual Block with a SE module on the input (equivalent to the EfficientNet architecture), and two transformer blocks: Block Attention and Grid Attention. Block Attention implements the ViT block applied to windows of predefined sizes. Grid Attention is more complicated; while the Block Attention module partitions the input feature maps into a set of four windows, the Grid Attention partition performs a more complicated operation that shuffles the pixels [63] of the feature maps according to the windows of the Block Attention module. All the attention blocks also include relative position embeddings [64]. The choice of MAxViT architecture is conditioned first in order to compare a ViT architecture with CNN architectures, and for its ability to extract the features at all five stages of the considered encoder framework, inherited from the SMP library. Meanwhile, the other transformer architectures skip the extraction of low-level features and extract only four stages of features, starting from the features reduced by the factor of four. Looking ahead, the extraction of low-level features is crucial for the segmentation of small objects.

### 3.5. Input Layer Adaptation

For input, the convolutional layer of the considered encoder neural networks was modified in the following way. The first convolutional layer of encoder neural network is usually adapted to process RGB images and has three input channels:

(1) $$W\in R^{N\times C\times h\times w},$$

where *N* is the number of output convolutional features, *C* is the number of input channels of the convolutional layer, and h and w are correspondingly the number of rows and columns in a convolutional kernel.

In order to adapt convolutional kernel weights to a new set of channels, we average the values of the existing weight tensor, then we concatenate the averaged weights $C_{ms}$ times ($C_{ms}$ is the number of multispectral channels):

$${\bar{W}}_{n,\;h,w}=\frac{1}{C}\sum_{i=1}^{C}W_{n,i,h,w},$$

$$W_{ms}=concatenate\left({\underline{W}}_{n,\;h,w}\times C_{ms}\right),$$

$$W_{ms}\in R^{N\times C_{ms}\times h\times w}.$$

This procedure provides an ability to process an arbitrary number of multispectral channels, while preserving the benefits of using pretrained encoder models.

### 3.6. Improving Small Object Segmentation

The structure of widespread convolutional and transformer neural networks for image processing implies the gradual reduction in spatial resolution of the processing feature maps with increasing the neural network depth. The first size reduction (usually, by half) is performed after the input convolutional layer or convolutional layer block. At the same time, the chosen encoder structure implies that the initial set of features are extracted after the first convolutional block. At the same time, multispectral satellite images from the Sentinel-2 database have a minimum spatial resolution of 10 m. Therefore, small objects on these images, such as roads, rivers, streams, ponds, clearings, etc., that may have sizes up to five pixels in the narrowest dimension, might be significantly reduced or even vanish from the feature maps after the first convolutional layer. Therefore, these reduced features may lose essential information about the objects, and the decoder of the neural network will not predict such objects correctly.

In order to overcome this limitation, we propose to manually set the stride of the input convolutional layer to one. This simple technique retains the size of low-level features and prevents the vanishing of small objects from the feature maps. On the other hand, this technique does not modify the values of the convolutional kernels. Therefore, it does not significantly break feature processing pipeline, where the consecutive layers are sensitive to the structure of feature maps produced on the previous. As a result, this technique can potentially improve the segmentation results of small objects. In this work, we consider two stride values: stride = 1 and stride = 2.

### 3.7. Experimental Setup

In our experiments, we utilized the following hardware and software setup. CPU: Intel Core i9-10900X; GPU RTX 2080 Ti 12 GB; DDR 16 GB; GNU Linux Debian 12; Python 3.12; pytorch 2.7.1; and CUDA 12.6. We utilize Adam [65] with parameters lr = 0.001, β_0_ = 0.9, and β_1_ = 0.999 as the optimization algorithm, and Cosine Annealing Warm restarts [66] as the learning rate scheduler with restart parameter T_0_ = 25 epochs. The batch size is set to 16. We utilize the following augmentation techniques of the training set: random horizontal and vertical flipping with the flipping probability of 0.5, and random affine transformation with a rotation range from 0 to 45 degrees, scale range from 0.7 to 1.5, shear range from 0 to 0.2, and translation range from 0 to 0.3. The pixels of the input images are Z-normalized to a mean value of 0 and standard deviation of 1 for all the processing channels independently. All the configurations of the models were trained for 300 epochs.

In order to train the models, we utilized unweighted multiclass cross-entropy (CE) loss adapted to segmentation:

$$CE\left(y,\;\hat{y}\right)=-\sum_{k=1}^{C}\sum_{i=1}^{H}\sum_{j=1}^{W}y_{ij}\log{\hat{y}}_{ij},$$

where W is the number of columns, H is the number of rows, C is the number of classes, ${\hat{y}}_{ij}$ is a predicted pixel of the segmentation mask, and $y_{ij}$ is a true segmentation mask pixel. We utilized the label smooth technique [67] with a smooth parameter equal to 0.15. Alternative functions (Weighted CE, Focal loss [68], Dice [69]) were tested in a limited pilot mode on the same spatially separated split, with identical hyperparameters, and showed lower IoU values on validation surfaces. Therefore, they were omitted from the main study, and in this paper, CE is retained as a stable reference point for comparing configurations.

Prevention of data leakage (spatial separation): division into train/test is performed at the level of entire geographic locations (10.5 × 10.5 km square), not at the level of patches (see Section 3.2). All patches (96 × 96) generated from one location belong exclusively to one set (train or test). In this way, there is no scenario where parts of the same scene or adjacent patches from the same area are found simultaneously in training and test, thus eliminating spatial “information leakage” and ensuring a realistic assessment of generalization to new terrains.

### 3.8. Assessment Metrics

In order to assess performance, we utilized the following metrics:

1. Intersection over Union (IoU): $IoU=\frac{TP}{TP\;+\;FP\;+\;FN},$
2. $Precision=\frac{TP}{TP\;+\;FP},$
3. $Recall=\frac{TP}{TP\;+\;FN},$

where *TP* is the number of true positive predictions, *TN* is the number of true negative predictions, *FP* is the number of false positive predictions, and *FN* is the number of false negative predictions.

We also computed the unweighted mean values of the metrics and the unweighted mean values of the metrics for applicable and non-applicable surfaces:

$$mMetric\;=\frac{1}{C}\sum_{i=1}^{C}{Metric\;}_{i},$$

where ${Metric\;}_{i}$ is IoU, precision, or recall values for a specific class *i*, and *C* is the number of all classes, or applicable or non-applicable classes, depending on the considering metric.

Taking into account the limited size of the dataset and the pronounced class imbalance, the obtained values of the evaluation metrics should be interpreted with a certain degree of caution. This paper shows the average values of IoU, precision, and recall metrics by classes and models, without explicit calculation of variance or confidence intervals. Therefore, small differences in performance between individual configurations, especially between different encoders, should not be viewed as statistically significant, but as indicative trends in model behavior.

## 4. Results

In Table 2, Table 3 and Table 4 the results of IoU, precision and recall metrics are presented. In all the tables, column names are interpreted as follows: Enc. means Encoder; Sp. represents the combination of Sentinel-2 spectral bands; Mean is an unweighted mean value of a considered metric across all the classes including unlabeled areas; N.A. Mean is a mean value of a non-applicable surface class metric; A. Mean is a mean value of an applicable surface class metric; Build. Represents building class; Transp is a transport class; N.A. Gnd. means non-applicable ground; N.A. Low B. means non-applicable low bushes; N.A. Wtl. is a non-applicable wetland class; A. Gnd. is applicable ground; A. Low B. is an applicable low bush class; A. Wtl. is an applicable wetland class; A. Wood is an applicable wood class; Ulbl is an unlabeled area. Bold font highlights the best results for each class and mean metric values within the encoder, while underlined font highlights the best metric results for all the experimental configurations.

**Figure 4:**
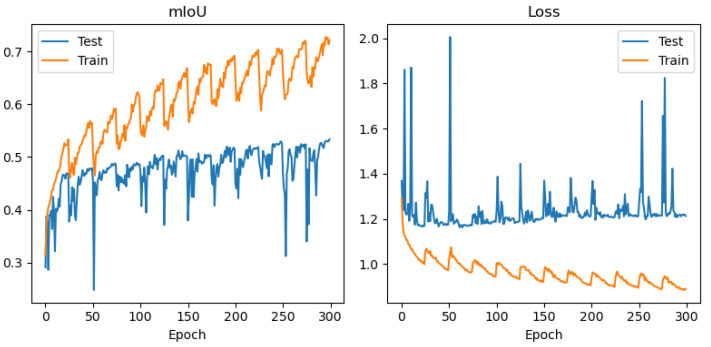
Training curves (mean IoU and loss function) for UNet with CSPDarknet53 encoder, RGB+NIR band processing, and input convolution stride = 1.

**Figure 5:**
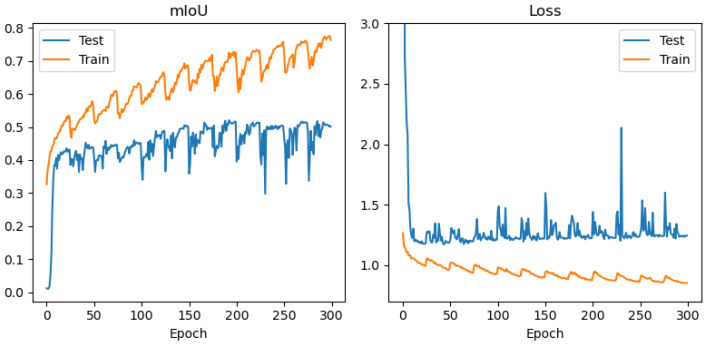
Training curves (mean IoU and loss function) for UNet with EfficientNetB2 encoder, RGB+NIR band processing, and input convolution stride = 1.

**Figure 6:**
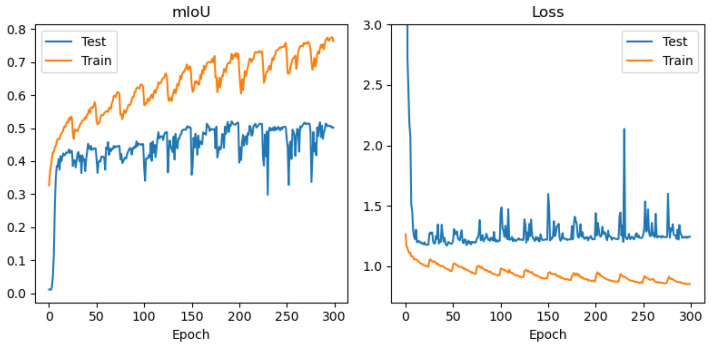
Training curves (mean IoU and loss function) for UNet with EfficientNetB2 encoder, with resolution of 10 m/pix band processing, and input convolution stride = 1.

**Figure 7:**
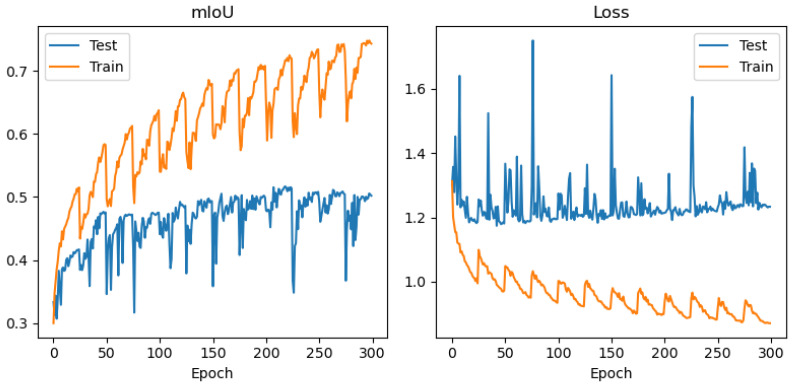
Training curves (mean IoU and loss function) for UNet with MAxViT encoder, RGB+NIR band processing, and input convolution stride = 2.

**Figure 8:**
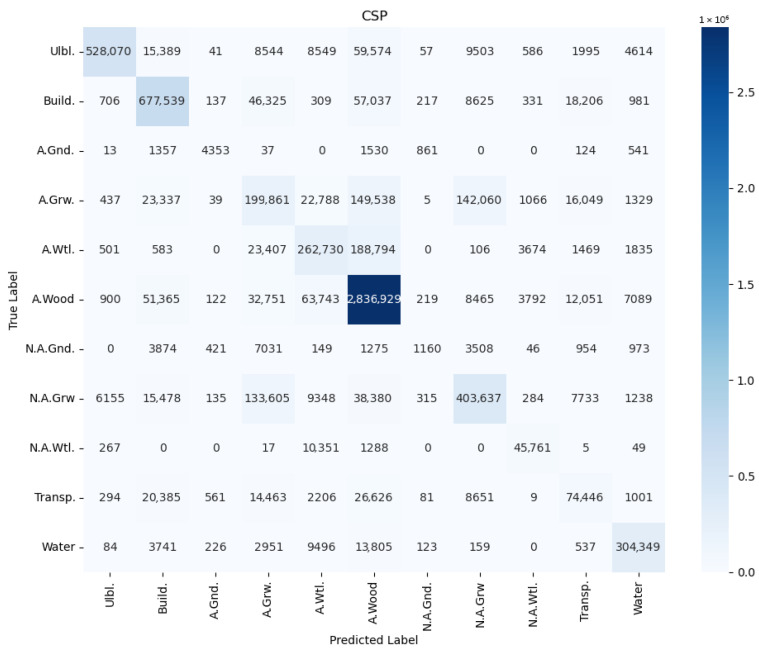
Confusion matrix for UNet with CSPDarkNet53 encoder, RGB+NIR band processing, and input convolution stride = 2 (CSP).

**Figure 9:**
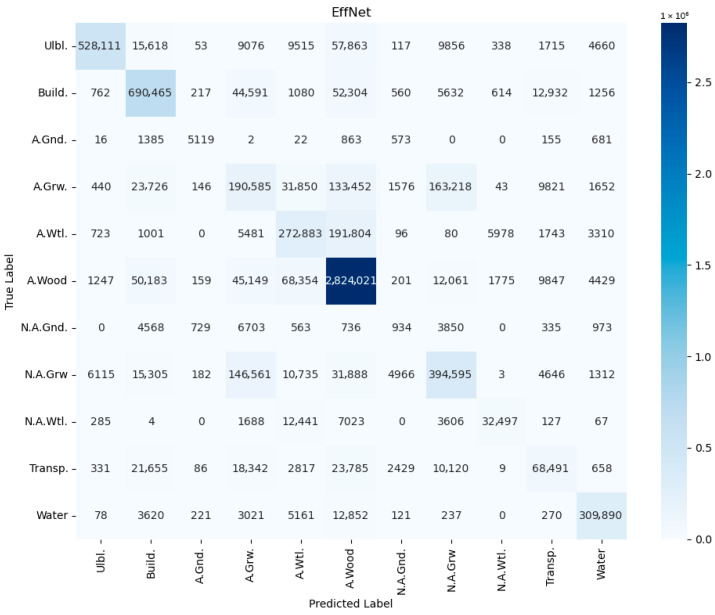
Confusion matrix for UNet EfficientNetB2 encoder, RGB+NIR band processing, and input convolution stride = 1 (EffNet).

**Figure 10:**
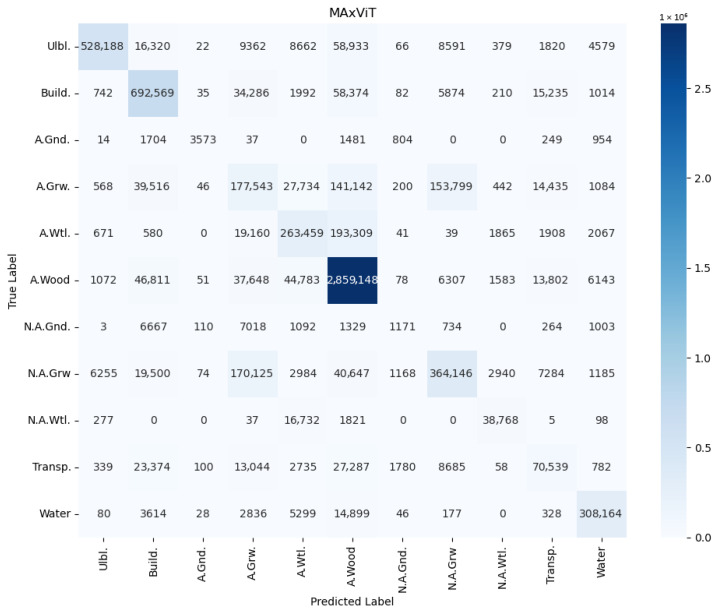
Confusion matrix for UNet with MAxViT encoder, RGB+NIR band processing, and input convolution stride = 2 (EffNet).

In order to avoid conclusions relying only on aggregated metrics (IoU/precision/recall), diagnostic displays of learning and errors were added to the paper: (i) learning curves (loss and mean IoU) for representative configurations (Figure 4, Figure 5, Figure 6 and Figure 7) and (ii) confusion matrices for the best configurations per mIoU (Figure 8, Figure 9 and Figure 10). These displays allow checking the stability of convergence, spotting potential overfitting, and identifying typical confounds between classes, thereby significantly increasing methodological transparency.

Figure 4, Figure 5, Figure 6 and Figure 7 demonstrate training curves of loss and mean IoU for CSP, EffNet, and MAxViT models correspondingly.

In order to perform detailed misclassification analysis, we provide confusion matrices (Figure 8, Figure 9 and Figure 10) for the best mIoU encoder configurations (Table 2). The presented results on confusion matrices show which class was misclassified with the other classes. The observed patterns are qualitatively consistent between the CSP, EfficientNet and MAxViT encoders. The in-depth discussion of misclassification is presented in Section 5.4.

### Visualization

Qualitative analysis of results represents an important addition to quantitative evaluation metrics, especially in the context of complex and class-imbalanced semantic segmentation tasks. The selection of the presented examples is not based on the “best cases”, but on spatially independent test locations (squares set aside for the test) and on typical scenes containing a combination of dominant and rare classes. The examples were chosen to cover (i) typical correct predictions on dominant classes, (ii) typical errors on boundaries between similar classes, and (iii) the impact of stride modification on thin/linear objects. Thus, qualitative presentations are used as a complement to metrics and confusion matrices, and not as an isolated “showcase”.

Visual displays allow a more detailed insight into the behavior of the model, identification of typical errors and a better understanding of the impact of certain architectural decisions on the quality of segmentation. This chapter presents representative examples of model predictions compared to benchmarks, as well as illustrations of typical failures and the effects of modifying the input convolutional stride.

**Figure 11:**
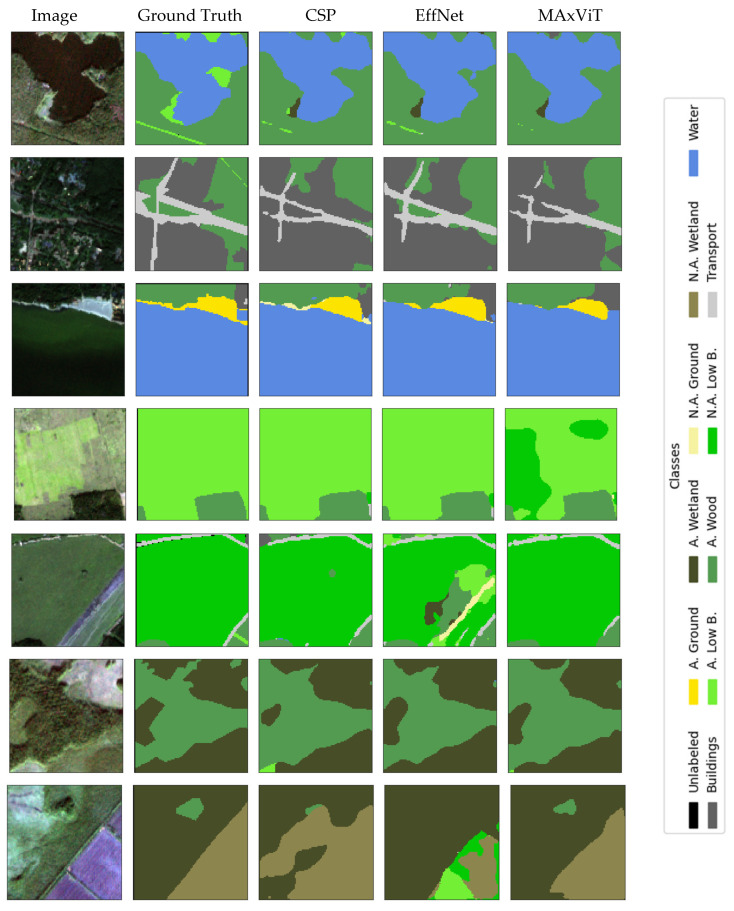
Visualization of UNet with CSPDarkNet53 encoder, RGB+NIR band processing, and input convolution stride = 2 (CSP); EfficientNetB2 encoder, RGB+NIR band processing, and input convolution stride = 1 (EffNet); MAxViT encoder, bands with 10 m spatial resolution processing, and input convolution stride = 2.

**Figure 12:**
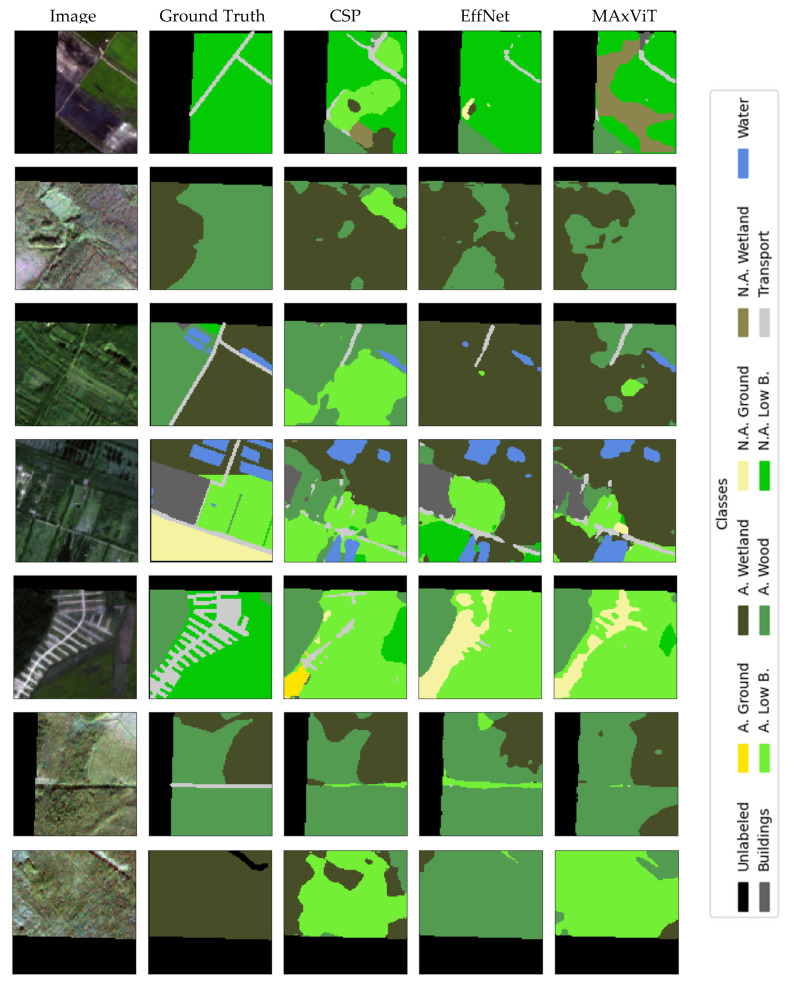
Visualization of segmentation errors of UNet with CSPDarkNet53 encoder, RGB+NIR band processing, and input convolution stride = 2 (CSP); EfficientNetB2 encoder, RGB+NIR band processing, and input convolution stride = 1 (EffNet); MAxViT encoder, bands with 10 m spatial resolution processing, and input convolution stride = 2.

**Figure 13:**
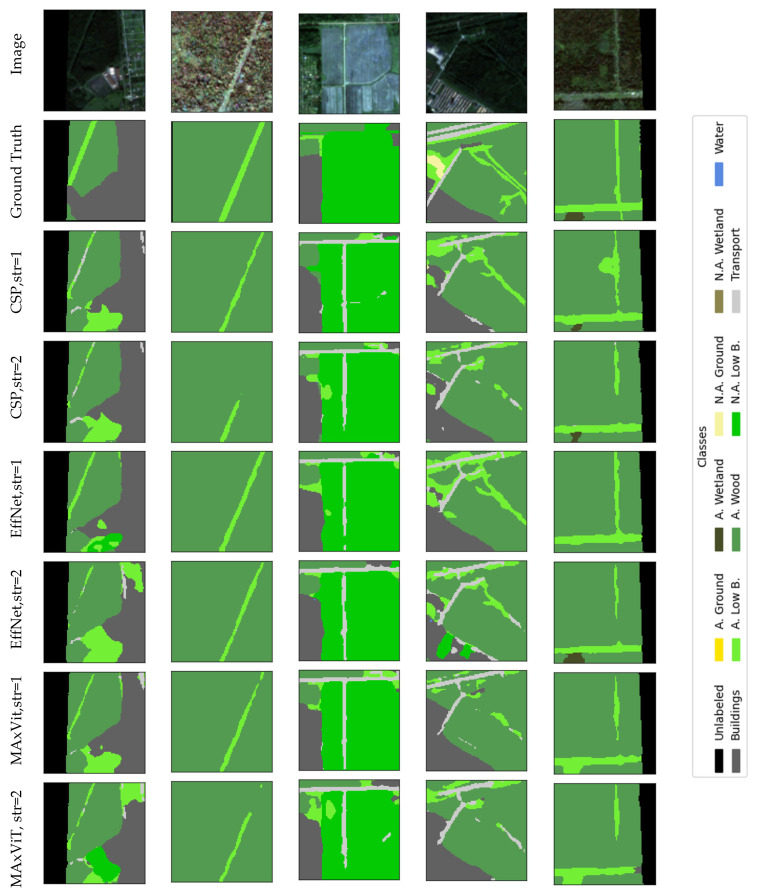
Visualization of stride = 1/stride = 2 impact for UNet with CSPDarkNet53 encoder, RGB+NIR band processing (CSP); EfficientNetB2 encoder, RGB+NIR band processing, (EffNet); MAxViT encoder, bands with 10 m spatial resolution processing.

In this section we provide a set of illustrations that visualize the results of the models with the best results of encoders. Figure 11 visualizes the segmentation of UNet with the CSPDarknet53 encoder, RGB+NIR bands, and input convolution stride = 2, which achieved the best mean IoU results (0.534); with the EfficientNetB2 encoder, RGB+NIR bands, and input convolution stride = 1, which achieved mean IoU results = 0.52; and with the MAxViT encoder, RGB+NIR bands, and input convolution stride = 2, which achieved mean IoU results = 0.517. Figure 12 visualizes the segmentation errors. Figure 13 visualizes stride = 1/stride = 2 impact.

**Figure 14:**
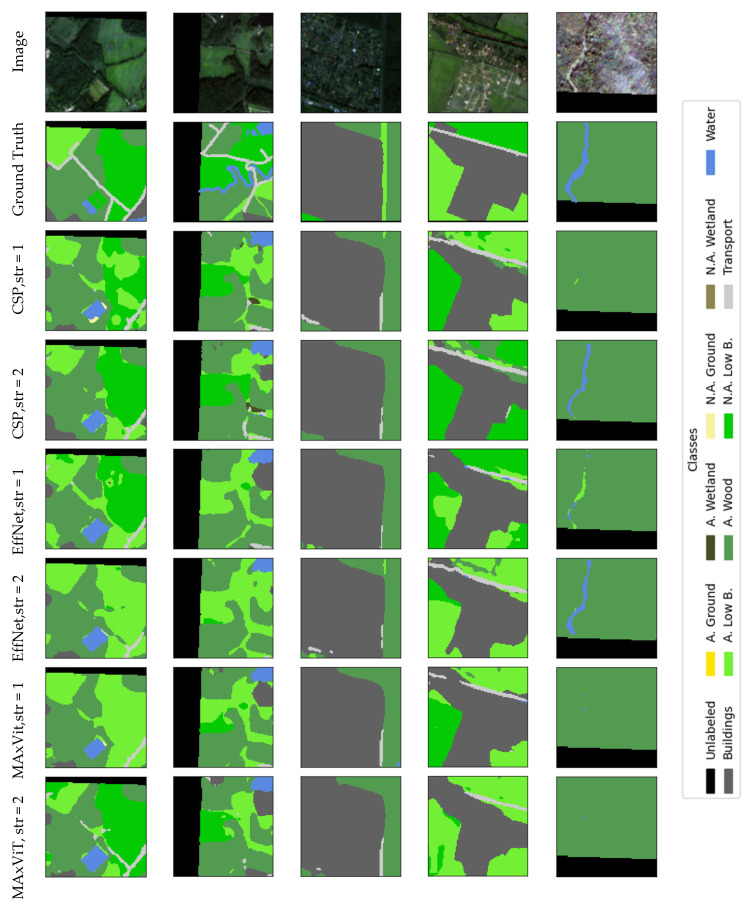
Visualization of stride = 1/stride = 2 segmentation errors for UNet with CSPDarkNet53 encoder, RGB+NIR band processing (CSP); EfficientNetB2 encoder, RGB+NIR band processing, (EffNet); MAxViT encoder, bands with 10 m spatial resolution processing.

The figures show a comparison of ground truth and model predictions for different encoder configurations. Visual analysis shows that stride = 1 can improve segmentation of thin and linear objects (especially transport and narrow clearings/lines within applicable low bushes), as more low-level detail is retained in early feature maps. The effect is not uniform across all classes and is not “dramatic” as is demonstrated in Figure 14; therefore, stride modification is treated as a simple, interpretable intervention that can bring local improvements, but does not solve all challenges (especially in rare classes and borderline cases).

## 5. Discussion

First of all, applicable surfaces are better segmented than non-applicable ones in general according to IoU and precision metrics. The best mean recall values for applicable and non-applicable classes are comparable. The best results in IoU, precision, and recall metrics were achieved by the classes of water, wood, and buildings. While the water class contains areas with low variations in pixel values, the wood class has the maximum number of pixels (Table 1), and buildings have the second highest number of labeled pixels. The worst results were achieved by non-applicable ground. Therefore, it is necessary to enlarge the labeled area for this class and/or perform internal differentiation between potential subclasses of this class (types of quarries in our case). Transport and applicable low bushes achieved relatively low results that can be explained by confusion with similar classes, such as buildings for the transport class and non-applicable low bushes for applicable low bushes. It is also interesting that non-applicable wetlands, in general, are segmented better than applicable wetlands.

The relatively low values of the average IoU in this work should be seen in the context of the complexity of the task itself and the pronounced class imbalance. Unlike standard land cover segmentation tasks, the functional suitability of surfaces for seismic installation is considered here, where the boundaries between classes are often not clearly defined and depend on a combination of spectral, spatial–structural and contextual factors. Particularly low performance for rare classes, such as “Applicable Ground” and “Non-applicable Ground”, is expected under conditions of extreme imbalance and limited spatial resolution of Sentinel-2 data. The results obtained represent a realistic baseline for this specific task and not an upper limit of possible performance.

Further, we perform in-depth comparisons of the encoder impact, processing of spectral band impact, and input stride impact, according to Section 3.1.

### 5.1. Encoder Impact

According to IoU results (Table 2), CSPDarknet53 outperforms the results of the EfficientNetB2 and MAxViT results for the most of the surface classes, except buildings, water, and applicable wood classes. The precision results (Table 3) give us a similar picture: CSPDarknet53 dominates the other models, except water, non-applicable wetlands, and all classes in general. The recall values (Table 4) also reproduce this picture, except buildings, water, and applicable wood.

The comparison of encoders shows a consistent dominance of CSPDarknet53 in most classes, while MAxViT does not show a stable advantage over CNN encoders in the observed configurations. In this paper, we do not introduce speculative explanations of the cause of that difference (e.g., “transformers require more data”), but limit the conclusion to empirical results obtained on the same training protocol and spatially independent test. Diagnostic learning curves (Figure 4, Figure 5, Figure 6 and Figure 7) do not indicate pronounced overfitting as a dominant problem but rather learning limitations in the given conditions (number of scenes + class imbalance + resolution), which is why MAxViT is treated in this paper as a valid, but not superior, baseline option compared to CNN encoders. Conclusions about MAxViT are strictly empirical and limited to a given dataset and the same training protocol: in the observed configurations, MAxViT does not show a consistent advantage over CNN encoders based classes and aggregated metrics, and the variations are interpreted as indicative trends, not as statistical evidence.

### 5.2. Spectral Band Impact

IoU results (Table 2) of CSPDarknet53 have shown that RGB bands dominate for applicable ground and applicable surfaces in general. RGB+NIR bands dominate transport, water, and applicable wood classes. Within stride = 1, CSPDarknet53 shows the best results for the full Sentinel-2 spectrum, while stride = 2 demonstrates RGB+NIR band domination. If we consider the precision metric (Table 3), we can notice RGB+NIR domination for the most of the classes (buildings, transport, water, applicable ground, applicable wetlands, non-applicable classes in general, all classes in general). We can also notice RGB+NIR domination in precision for both stride = 1 and stride = 2. However, recall (Table 4) gives a different picture: RGB+NIR dominates only for non-applicable wetland and applicable wood classes. Regarding differences within the same stride values, stride = 1 demonstrates the best recall values for the full Sentinel-2 spectrum, while stride = 2 demonstrates the best recall values for RGB+NIR bands. As a result, for CSPDarknet53, RGB+NIR spectral bands show better results compared to the other bands.

IoU results (Table 2) of *EfficientNetB2* have shown that RGB+NIR bands dominate only for non-applicable surfaces in general, while spectral bands with 10 and 20 m/pix spatial resolution dominate in applicable ground, applicable low bushes, and applicable surfaces in general. Considering IoU class results within the same stride values, EfficientNetB2 tends to achieve better results for 10 and 20 m/pix spatial resolution bands for stride = 1 and RGB+NIR bands for stride = 2. Precision (Table 3) demonstrates that RGB+NIR bands also dominate at non-applicable surfaces in average segmentation. However, RGB+NIR also demonstrates the best results for all strides in non-applicable ground and building class segmentation. At the same time, 10 and 20 m/pix spatial resolution bands dominate in non-applicable low bush segmentation, according to precision results. EfficientNetB2 precision results demonstrate a similar picture for the results within the same stride: 10 and 20 m/pix spatial resolution bands and the full spectrum outperform the other bands, while RGB and RGB+NIR bands outperform the other band combinations. At the same time, recall values of the EfficientNetB2 encoder (Table 4) demonstrate the domination of RGB bands both for distinct classes (buildings, transport, applicable ground, all classes in general), within stride = 1, and within stride = 2. As a result, RGB+NIR demonstrates the best IoU and precision for EfficientNetB2; however, recall tends to achieve the best results for RGB bands. Therefore, we can conclude that a small number of input channels is more preferable for the EfficientNetB2 encoder.

Regarding the *MAxViT* model, its IoU results (Table 2) demonstrate that the full spectrum and 10 and 20 m/pix spatial resolution bands outperform the other band combinations for stride = 1, while RGB and RGB+NIR bands outperform the other combinations for stride = 2. At the same time, IoU does not reveal the best spectral band combinations for the distinct classes. A similar picture is demonstrated in terms of precision (Table 3) for the stride = 1 and stride = 2 comparison. However, RGB+NIR provides the best precision results for applicable ground, applicable classes in general, and all classes in general. RGB demonstrates the best precision for buildings, and the bands with spatial resolution of 10 and 20 m/pix for non-applicable growth. MAxViT recall results (Table 4) also reproduce the picture for a stride = 1 and stride = 2 comparison. Recall results reveal the best combination for non-applicable growth only.

Changing the stride in the first convolutional layer affects the effective spatial resolution of the feature maps that are passed to the pre-trained encoders. Although this modification deviates from the original configuration used during ImageNet pretraining, experimental results show that CNN-based encoders retain the ability to adapt and achieve improved preservation of thin and linear structures relevant to seismic applications. This trade-off between strict compatibility with overtraining and better preservation of local details was considered consciously, keeping in mind the operational requirements of the task.

Combining all the results together, we can conclude that the best results were achieved by the models that process the RGB+NIR band in general. This is particularly true for CNN encoders. At the same time, the comparative analysis of the various spectral band combinations did not reveal the combinations that can characterize a certain class of the considered surfaces.

The results indicate that the choice of encoders and spectral ranges has a greater impact on the performance of the model than individual architectural modifications, while the change in stride represents an efficient but targeted strategy for improving the segmentation of smaller objects. The obtained findings confirm that the considered task is inherently demanding and that it requires careful balancing between models, data and evaluation criteria.

### 5.3. Input Convolution Stride Impact

IoU results (Table 2) have shown that for *CSPDarknet53*, stride = 1 achieves the best results for transport, applicable ground, applicable wood, and applicable surfaces in general, while stride = 2 dominates for applicable low bushes and non-applicable wetlands. It is notable that the best mean IoU for all the experiments is achieved by the configuration with stride = 2 and RGB+NIR multispectral bands. Precision results (Table 3) have shown that stride = 1 dominates in transport, water, and applicable wetland segmentation for all the bands. Stride = 2 dominates in non-applicable wetlands also for all the bands. It is notable that stride = 2 dominates in RGB+NIR bands for precision results of the most classes. It is also notable that the best mean precision was achieved with the same stride and band configuration as IoU (stride = 2, RGB+NIR). Recall results (Table 4) have shown that stride = 1 dominates for applicable surfaces in general, transport, applicable ground, applicable low bushes, and applicable wetlands. At the same time, stride = 2 did not achieve the best results for all the bands. However, it dominates in water and non-applicable low bush segmentation. It is also notable that stride = 2 dominates in processing RGB+NIR bands. Best mean recall for all the experiments was achieved by CSPDarknet53, which processes all the Sentinel-2 bands, and stride = 1.

IoU results (Table 2) have shown that for *EfficientNEtB2*, stride = 1 outperforms stride = 2 for most of the classes and processing bands. The same tendencies demonstrate precision (Table 3) and recall (Table 4) results for EfficientNetB2. It is also worth noting that the best precision value for non-applicable surfaces in general was demonstrated by EfficientNetB2 (RGB+NIR bands and stride = 1).

IoU results (Table 2) have shown that for *MAxVit*, stride = 2 dominates in non-applicable low bushes and non-applicable wetlands. Stride = 2 also outperforms stride = 1 in average and for non-applicable surfaces segmentation according to IoU metric. It is notable that the transport class for RGB and 10–20 m spatial resolution bands and stride = 2 outperforms stride = 1, while CNN models demonstrate s strong domination of stride = 1 in transport class segmentation. Speaking of stride’s impact within the same spectral bands, stride = 2 outperforms stride = 1 in all the bands, except the processing of all the spectral bands. Precision (Table 3) demonstrates a similar picture; however, it differs in its details. Stride = 2 dominates for all the bands in non-applicable low bush segmentation only and dominates for most of the bands for all classes in general, as well as water, non-applicable ground, non-applicable wetlands, and applicable wetlands. At the same time, stride = 1 dominates in buildings, transport, applicable low bushes, applicable wood, and non-applicable surface segmentation in general. Speaking of stride’s impact within the same spectral bands, precision for stride = 2 outperforms stride = 1 for RGB and RGB+NIR channels, as well as for the IoU metric. However, stride = 1 outperforms stride = 2 for 10–20 m spatial resolution bands, as well as for full Sentinel-2 spectrum bands. It is also worth noting that the best precision value for applicable surfaces in general was demonstrated by MAxViT (RBG+NIR bands and stride = 2). Recall (Table 3) demonstrates a different picture. Neither of the classes demonstrated stride = 2 domination for all the spectral band combinations (however, stride = 2 dominates for the most of the bands for non-applicable low bushes, non-applicable wood, and non-applicable surfaces in general), while stride = 1 dominates for applicable low bushes, applicable wetlands, applicable surfaces in general (for all the spectral bands), buildings, transport, water, non-applicable ground, non-applicable wetlands, applicable low bushes, and all the classes in general (for the most of the spectral bands).

Generally speaking, stride = 1 demonstrates segmentation effectiveness improvement for small objects (class transport in our case), especially for CNN encoders. The other classes that are affected by stride = 1 are applicable surfaces: wood, ground, and wetlands. It is also notable that stride = 1 dominates in the most of the classes during the processing of bands with 10–20 m spatial resolution (except the MAxViT model), as well as during full spectrum processing. The MAxVit model demonstrates that stride value modification mostly decreases the segmentation results for most of the processing spectral bands (according to IoU and precision metrics). Therefore, we could propose that ViT architectures depend less on the granularity of the initial feature maps.

Differences in sensitivity to stride modification between the CNN encoder and MAxViT are considered in this paper as empirical observations: stride = 1 has a clearer and more consistent effect on transport and other thin/linear structures with the CNN encoder, while with MAxViT this effect is not stable across all combinations of channels and metrics. In this paper, no “strong” conclusions are drawn about the internal mechanisms of the transformer (tokenization/attention), because this requires additional diagnostics (e.g., attention map analysis) that are not the goal of the baseline study; instead, the finding is formulated as a practical recommendation within the observed protocol and dataset.

### 5.4. Misclassification Analysis

Misclassifications depicted in confusion matrices in Figure 8, Figure 9 and Figure 10 and visually illustrated in Figure 12 and Figure 14 substantiate the need for analysis, which classes are confused, and how these confusions could be resolved. Here the most notable cases are discussed. Building territory includes industrial, urban, and rural areas. The misclassification of buildings with applicable wood, applicable growth, and transport classes substantiates the need for distinction of rural building areas in order to address misclassification with growth and wood classes as well the need for refinement of transport class areas. Applicable wetlands are heavily misclassified with applicable wood areas. It is worthwhile to separate intersections of wood and wetland areas from bare wood and bare wetlands to a new class of wetland wood. The most misclassified class is non-applicable ground. Misclassification of non-applicable ground with non-applicable growth substantiates the necessity of distinction of agricultural bare ground from agricultural growth. Wood cutting areas should be distinct from the applicable growth class due to applicable growth being misclassified with applicable wood. Another reason is an enlargement of the list of small and linear objects. Figure 13 shows that cutting areas are linear objects like transport objects. The transport class, in turn, contains railways, paved and unpaved roads, roads, and roads in woods and urban territory. Distinguishing all the mentioned subclasses could potentially improve segmentation performance.

### 5.5. Limitations

Several important limitations of this study should be acknowledged. First, the dataset is geographically restricted: all scenes originate from the Saint Petersburg region, which limits environmental, climatic and land-use variability and therefore constrains the external validity of the models beyond this area. This issue includes the issue of extreme class imbalance: a few classes (e.g., “Applicable wood”) dominate the pixel count while several valuable classes (e.g., “Applicable ground”) are scarce. These data are described in Section 3.2.

Second, the misclassification analysis (Section 5.4, Figure 8, Figure 9, Figure 10, Figure 11, Figure 12 and Figure 13) revealed the limitations in the proposed classification (Figure 2) of applicable surfaces for seismic sensor installation. This limitation includes the need not only for the strict enlargement of the dataset but also of developing a labeling procedure with fine-grained surface classification. This procedure, in turn, implies separation of a general class into a set of subclasses (e.g., transport into paved/unpaved roads, railroads; buildings into industrial, rural, and urban areas, distinguishing cited areas in the wood, etc.). However, relabeling the semantic segmentation georeferenced dataset is a labor-intensive process, and therefore it will be performed during further research.

Third, the input imagery is limited by the native spatial resolution of Sentinel-2 (10 m/pixel for the highest-resolution bands). Many features relevant to seismic sensor installations (narrow tracks, small clearings, fine linear disturbances) are sub-pixel or occupy only a few pixels at this scale, which inevitably reduces detectability and contributes to lower IoU and recall for small/linear classes. This resolution constraint also complicates accurate boundary delineation and increases confusion between spectrally similar classes. However, the 10 m/pix resolution of Sentinel-2 images is appropriate for the initial survey of large areas of the terrain for seismic sensor installation. Another reason for Sentinel-2 satellite imagery choice is the wide radiation spectral range of its images. The impact of using extra spectral ranges is itself of research interest in the task of terrain survey for seismic sensor installation. Another reason for using Sentinel-2 imagery is its availability for arbitrary geographical locations and shooting dates using the Sentinel Hub service https://www.sentinel-hub.com/develop/api/ (accessed on 19 December 2025).

Finally, in this work, we focused more on the aspects of multispectral data processing using different encoders and other preprocessing techniques. Therefore, we utilized only one UNet architecture that is informative as a baseline. However, we admit that this approach does not cover the other modern segmentation architectures (e.g., DeepLabV3+, encoder–decoder transformers, or ensemble methods that might improve robustness). The above limitations affect classes and metrics asymmetrically. Geographical locality limits external validity (model can learn regional textures/structures); Sentinel-2 resolution disproportionately affects thin and sub-pixel objects (transport and narrow clearings), while class imbalance leads to “conservative” predictions favoring dominant classes. The aggregated mean results are used as an indicator of the general behavior of the model, while the performance based on rare classes is interpreted cautiously and with the support of confusion matrices and qualitative analysis.

### 5.6. Generalizations of the Results

As a result, we can conclude that the clearest and most obvious impact for the resulting segmentation is achieved by the CSPDarkNet53 encoder. Therefore, it is worthwhile to consider the other factors regarding the encoder. Indeed, while the impact of the processing bands and the input convolutional stride may significantly vary, the comparison of the encoder shows more clear differences. It is also worth noting that utilizing the ViT model (MAxViT in our case) did not bring us segmentation enhancement. This could potentially be explained by the fact that the transformer-based models in general (including ViT) often require more data for training than CNNs [70,71,72].

The comparison of the processing spectral band combinations also reveals important tendencies. The comparison results have shown that utilizing the RGB+NIR band combination (these bands have 10 m/pix spatial resolution) achieves better segmentation results in general than the other band combinations. Therefore, this combination offers the most perspective for the semantic segmentation of applicable surfaces for seismic sensor installation. However, the extension of the dataset could potentially increase the performance of a spectral band combination with many bands.

The input convolutional stride = 1 has more impact for linear object and small object segmentation, while the objects that have large squares (wood, water, etc.) are more sensitive to stride = 2. The other interesting tendency is that stride = 1 becomes more valuable for processing the bands with 10 and 20 m/pix spatial resolution, as well as for processing the full Sentinel-2 spectrum.

Misclassification analysis substantiated the need for both refining of labeling of the existent dataset and distinguishing the subclasses that could potentially reduce misclassification rate (e.g., bare agricultural ground from applicable growth).

## 6. Conclusions

This study addressed the novel task of semantically segmenting surfaces applicable for seismic sensor installation using multispectral Sentinel-2 imagery and the U-Net architecture. Through a comprehensive empirical evaluation, we established a performance baseline and investigated the impact of key model components. The results clearly indicate that the choice of encoder has the most pronounced effect on segmentation performance. The convolutional CSPDarknet53 encoder consistently outperformed both EfficientNetB2 and the transformer-based MAxViT model across most surface classes and metrics. Within this dataset and identical training protocol, MAxViT did not show a consistent advantage over CNN encoders; therefore, this finding is formulated as an empirical conclusion limited to the observed conditions, without speculative attribution of causes (e.g., “need for a larger dataset”). Regarding spectral input, the combination of RGB and NIR bands, all at 10 m/pixel spatial resolution, was identified as the most effective configuration for this task. This combination provided a favorable balance between spectral information and spatial detail, generally surpassing both simpler (RGB) and more complex (full 13-band) inputs. Modifying the stride of the input convolutional layer to 1 shows a tendency to improve the segmentation of thin and linear objects (e.g., transport), especially with CNN encoders. The effect is not uniform across all classes and configurations, so stride = 1 is recommended as a targeted, interpretable intervention for fine details, rather than a one-size-fits-all solution. In the context of baseline evaluation and observed configurations, the most stable choice is U-Net with the CSPDarknet53 encoder and RGB+NIR input (10 m), while stride = 1 can be considered when preserving thin/linear structures is a priority.

It is necessary to perform more detailed labeling of the dataset, including distinction of subclasses discussed in Section 5.4 (e.g., distinguishing subclasses of paved/unpaved roads, railroads, etc., from transport class). Another potential research direction is a multimodal approach that integrates satellite imagery with digital elevation models (DEMs) and derived topographic attributes (e.g., slope, aspect, curvature). Elevation-informed features may help disambiguate spectrally similar classes and better represent terrain constraints relevant to seismic sensor deployment, particularly in forested or heterogeneous landscapes. The next research direction is focused on exploring more advanced segmentation architectures, and investigating the fusion of spectral and spatial features to enhance the accuracy and robustness of the search for applicable locations for seismic sensor nodal system installation. Temporal information from Sentinel-2 time series will be incorporated to assess temporal consistency and exploit phenological and land-use dynamics. Recurrent or temporal-attention-based architectures may enable the model to distinguish persistent structural features from transient artifacts such as seasonal vegetation changes or temporary disturbances. To address persistent class imbalance, hard-negative mining and class-aware sampling strategies will be evaluated, with particular emphasis on improving detection of low-frequency but operationally critical classes. By explicitly focusing training on confusing background regions and rare-class errors, these strategies may enhance minority-class recall without degrading overall performance.

## Figures and Tables

**Table 1:** Dataset of applicable surfaces for seismic sensor installation statistics.

Surface Class	Number of Pixels, M	Area Portion, %	Area Portion Train, %	Area Portion Test, %
Buildings	2.75	12.9	13.1	12.6
Transport	0.54	2.6	2.7	2.3
Water	1.07	5.0	5.9	5.4
Non-applicable Ground	0.14	0.6	0.7	0.3
Non-applicable Low Bushes	1.95	9.2	9.0	9.6
Non-applicable Wetlands	0.13	0.6	0.5	0.9
Applicable Ground	0.05	0.2	0.3	0.1
Applicable Low Bushes	1.91	9.0	9.1	8.7
Applicable Wetlands	1.61	7.6	7.6	7.5
Applicable Wood	10.23	48.2	48.2	48.0
Unlabeled Area	0.87	4.1	3.9	4.6

**Table 2:** IoU UNet results. Bold font highlights the best results for each class and mean metrics values within the encoder, while underlined font highlights the best metric results for all the experimental configurations.

Enc	Sp	Stride	Mean	N.A. Mean	A. Mean	Build.	Transp.	Water	N.A. Gnd.	N.A. Low B.	N.A. Wtl.	A. Gnd.	A. Low B.	A. Wtl	A. Wood	Ulbl.
CSP	RGB	1	0.513	0.474	0.497	0.722	0.353	0.76	0.038	0.459	0.511	**0.529**	0.234	0.434	0.79	**0.817**
		2	0.501	0.486	0.446	0.719	0.334	0.761	**0.068**	0.489	0.543	0.392	0.204	0.406	0.781	**0.817**
	10 m	1	0.528	0.509	0.484	0.726	**0.374**	**0.86**	0.038	0.479	0.578	0.46	0.234	0.443	**0.799**	**0.817**
		2	**0.534**	**0.528**	0.471	0.716	0.358	0.857	0.055	**0.506**	**0.678**	0.415	0.242	0.431	0.798	**0.817**
	10–20 m	1	0.513	0.488	0.474	0.717	0.365	0.823	0.052	0.47	0.5	0.431	**0.247**	0.421	0.798	**0.817**
		2	0.503	0.484	0.452	0.721	0.352	0.817	0.029	0.483	0.504	0.397	0.188	0.429	0.795	**0.817**
	All	1	0.529	0.51	**0.486**	**0.729**	0.368	0.831	0.062	0.495	0.576	0.454	0.242	**0.451**	0.797	**0.817**
		2	0.507	0.482	0.467	0.686	0.35	0.764	0.043	0.456	0.596	0.445	0.214	0.427	0.782	**0.817**
EffNet	RGB	1	0.499	0.472	0.462	0.71	0.355	0.742	0.017	0.363	0.644	0.424	0.232	0.408	0.782	**0.817**
		2	0.494	0.475	0.442	0.716	0.314	0.752	0.012	0.444	0.609	0.414	0.187	0.373	0.792	0.816
	10 m	1	0.519	**0.504**	0.468	0.713	0.336	0.872	0.02	0.436	**0.648**	0.448	0.233	0.402	0.789	0.816
		2	0.513	0.498	0.461	0.72	0.339	0.85	0.008	0.435	0.633	0.417	0.226	0.411	0.79	0.816
	10–20 m	1	**0.52**	0.493	**0.487**	0.729	**0.36**	**0.874**	0.031	0.478	0.489	**0.483**	0.228	0.436	0.8	0.816
		2	0.495	0.461	0.467	0.715	0.315	0.842	0.015	0.454	0.426	0.415	**0.246**	0.418	0.789	0.816
	All	1	0.512	0.491	0.466	**0.73**	0.354	0.844	**0.053**	0.469	0.495	0.384	0.245	0.438	**0.799**	**0.817**
		2	0.502	0.477	0.462	0.72	0.328	0.813	0.027	**0.485**	0.488	0.381	0.229	**0.446**	0.792	**0.817**
MaxViT	RGB	1	0.492	0.455	0.468	0.726	0.33	0.74	0.029	0.415	0.489	0.447	0.194	0.432	0.799	0.816
		2	0.493	0.467	0.453	0.729	0.333	0.755	0.026	0.422	0.535	0.417	0.214	0.385	0.795	0.816
	10	1	0.509	0.492	0.459	0.727	**0.361**	0.856	0.045	0.432	0.528	0.372	0.217	0.444	0.802	**0.817**
		2	**0.517**	**0.505**	0.46	0.715	0.346	0.87	**0.05**	0.455	**0.594**	0.385	0.209	0.443	**0.804**	0.816
	10–20 m	1	0.502	0.477	0.464	0.722	0.327	0.86	0.034	0.437	0.482	**0.465**	0.221	0.383	0.79	0.798
		2	0.509	0.487	0.466	0.717	0.342	0.862	0.003	0.449	0.547	0.414	0.22	0.435	0.797	**0.817**
	All	1	0.514	0.49	**0.476**	**0.732**	0.335	**0.874**	0.006	0.457	0.534	0.405	**0.225**	**0.468**	**0.804**	**0.817**
		2	0.508	0.491	0.456	0.72	0.334	0.856	0.025	**0.462**	0.55	0.343	0.212	0.465	0.802	**0.817**

**Table 3:** Precision UNet results. Bold font highlights the best results for each class and mean metrics values within the encoder, while underlined font highlights the best metric results for all the experimental configurations.

Enc	Sp	Stride	Mean	N.A. Mean	A. Mean	Build.	Transp.	Water	N.A. Gnd.	N.A. Low B.	N.A. Wtl.	A. Gnd.	A. Low B.	A. Wtl	A. Wood	Ulbl.
CSP	RGB	1	0.679	0.65	0.647	0.839	0.573	0.936	0.132	0.662	0.755	0.748	0.38	0.615	0.846	0.982
		2	0.672	0.664	0.607	0.848	0.565	0.885	0.186	0.658	0.843	0.674	0.361	0.54	0.851	**0.983**
	10 m	1	0.697	0.683	0.646	**0.86**	**0.594**	**0.953**	0.179	0.664	**0.847**	0.702	0.402	0.635	0.845	**0.983**
		2	**0.716**	**0.704**	**0.666**	0.833	0.557	0.939	**0.382**	**0.69**	0.824	0.721	**0.426**	**0.674**	0.841	**0.983**
	10–20 m	1	0.692	0.66	**0.666**	0.82	0.577	0.924	0.196	**0.69**	0.755	**0.797**	0.389	0.622	0.855	0.982
		2	0.674	0.637	0.653	0.822	0.57	0.898	0.105	0.631	0.795	0.752	0.379	0.636	0.847	0.982
	All	1	0.686	0.664	0.645	0.851	0.573	0.901	0.195	0.688	0.776	0.703	0.417	0.605	**0.856**	0.982
		2	0.67	0.635	0.644	0.82	0.558	0.856	0.176	0.658	0.741	0.736	0.366	0.632	0.839	0.981
EffNet	RGB	1	0.644	0.625	0.588	0.822	0.545	0.894	0.052	0.656	0.783	0.588	0.368	0.551	0.844	0.982
		2	0.664	0.639	0.623	0.798	0.546	**0.948**	0.049	0.63	0.863	0.677	0.351	0.63	0.834	0.981
	10 m	1	**0.707**	**0.71**	0.636	**0.847**	0.596	0.944	**0.309**	0.655	**0.906**	0.705	0.374	0.629	0.835	0.979
		2	0.692	0.684	0.63	0.807	0.551	0.93	0.263	0.68	0.876	0.684	0.388	0.608	0.842	0.98
	10–20 m	1	0.686	0.654	**0.662**	0.834	**0.622**	0.942	0.081	0.654	0.788	**0.741**	0.404	**0.657**	0.846	0.981
		2	0.654	0.608	0.64	0.825	0.566	0.909	0.078	**0.706**	0.562	0.686	0.411	0.629	0.836	0.981
	All	1	0.692	0.676	0.645	0.834	0.61	0.947	0.177	0.695	0.791	0.698	0.407	0.631	0.844	**0.983**
		2	0.675	0.658	0.625	0.82	0.552	0.895	0.163	0.676	0.84	0.625	**0.418**	0.61	**0.848**	0.981
MaxViT	RGB	1	0.664	0.638	0.622	**0.855**	0.569	0.916	0.096	0.599	0.794	0.699	0.343	0.601	0.847	0.98
		2	0.674	0.656	0.625	0.813	0.556	**0.951**	0.14	0.64	0.834	0.646	0.368	0.654	0.833	0.98
	10	1	0.688	0.66	0.658	0.816	0.58	0.944	0.107	**0.67**	0.844	0.766	0.377	0.639	0.848	0.982
		2	**0.711**	**0.672**	**0.701**	0.814	0.56	0.942	**0.215**	0.664	0.838	**0.885**	0.377	**0.702**	0.841	0.981
	10–20 m	1	0.681	0.67	0.63	0.85	0.591	0.935	0.058	0.651	**0.937**	0.6	**0.407**	0.698	0.818	0.946
		2	0.674	0.644	0.642	0.822	0.584	0.943	0.015	0.666	0.836	0.689	0.376	0.659	0.842	0.981
	All	1	0.672	0.664	0.606	0.838	**0.621**	0.943	0.042	0.655	0.886	0.569	0.39	0.604	**0.86**	**0.983**
		2	0.686	0.665	0.644	0.829	0.571	0.922	0.086	0.653	0.932	0.658	0.387	0.687	0.843	0.981

**Table 4:** Recall UNet results. Bold font highlights the best results for each class and mean metrics values within the encoder, while underlined font highlights the best metric results for all the experimental configurations.

Enc	Sp	Stride	Mean	N.A. Mean	A. Mean	Build.	Transp.	Water	N.A. Gnd.	N.A. Low B.	N.A. Wtl.	A. Gnd.	A. Low B.	A. Wtl	A. Wood	Ulbl.
CSP	RGB	1	0.613	0.563	**0.635**	0.838	0.478	0.802	0.05	0.599	0.613	**0.643**	0.613	0.563	**0.635**	0.838
		2	0.603	0.579	0.582	0.825	0.45	0.845	**0.097**	0.655	0.604	0.484	0.603	0.579	0.582	0.825
	10 m	1	0.622	0.591	0.615	0.823	0.503	0.898	0.046	0.633	0.645	0.572	0.622	0.591	0.615	0.823
		2	0.629	**0.625**	0.584	0.836	0.501	0.907	0.06	0.655	**0.793**	0.494	0.629	**0.625**	0.584	0.836
	10–20 m	1	0.609	0.582	0.594	0.851	0.498	0.883	0.066	0.596	0.596	0.484	0.609	0.582	0.594	0.851
		2	0.598	0.588	0.556	**0.854**	0.481	0.9	0.039	**0.674**	0.579	0.457	0.598	0.588	0.556	**0.854**
	All	1	**0.635**	0.612	0.622	0.836	**0.507**	**0.915**	0.084	0.638	0.692	0.562	**0.635**	0.612	0.622	0.836
		2	0.614	0.595	0.589	0.807	0.485	0.876	0.053	0.597	0.753	0.53	0.614	0.595	0.589	0.807
EffNet	RGB	1	**0.614**	0.569	**0.628**	0.839	**0.504**	0.813	0.024	0.448	**0.784**	**0.603**	**0.614**	0.569	**0.628**	0.839
		2	0.584	0.563	0.555	**0.874**	0.425	0.785	0.016	0.601	0.674	0.516	0.584	0.563	0.555	**0.874**
	10 m	1	0.607	0.576	0.599	0.819	0.434	0.92	0.02	0.565	0.695	0.552	0.607	0.576	0.599	0.819
		2	0.607	**0.583**	0.589	0.869	0.468	0.909	0.008	0.548	0.696	0.517	0.607	**0.583**	0.589	0.869
	10–20 m	1	0.613	0.581	0.606	0.852	0.461	**0.924**	0.048	**0.64**	0.563	0.581	0.613	0.581	0.606	0.852
		2	0.6	0.565	0.595	0.843	0.415	0.919	0.019	0.559	0.638	0.512	0.6	0.565	0.595	0.843
	All	1	0.602	0.572	0.592	0.855	0.458	0.886	**0.07**	0.591	0.57	0.461	0.602	0.572	0.592	0.855
		2	0.601	0.567	0.594	0.855	0.446	0.899	0.032	0.632	0.538	0.494	0.601	0.567	0.594	0.855
MaxViT	RGB	1	0.588	0.539	0.6	0.828	0.439	0.794	0.04	0.575	0.56	0.553	0.588	0.539	0.6	0.828
		2	0.585	0.55	0.577	**0.877**	0.454	0.786	0.031	0.553	0.598	0.541	0.585	0.55	0.577	**0.877**
	10	1	0.599	0.578	0.572	0.87	**0.49**	0.902	0.073	0.549	0.585	0.42	0.599	0.578	0.572	0.87
		2	0.602	**0.595**	0.554	0.855	0.474	0.919	0.06	0.591	**0.671**	0.405	0.602	**0.595**	0.554	0.855
	10–20 m	1	0.596	0.551	0.604	0.827	0.424	0.915	**0.075**	0.57	0.498	**0.673**	0.596	0.551	0.604	0.827
		2	0.599	0.568	0.589	0.849	0.451	0.91	0.004	0.58	0.613	0.509	0.599	0.568	0.589	0.849
	All	1	**0.613**	0.563	**0.633**	0.853	0.421	**0.922**	0.007	0.601	0.574	0.585	**0.613**	0.563	**0.633**	0.853
		2	0.594	0.572	0.567	0.846	0.447	**0.922**	0.035	**0.612**	0.573	0.418	0.594	0.572	0.567	0.846

## Data Availability

The data presented in this study are available for research purposes at https://drive.google.com/drive/folders/1RDPzZn3LJyp0j8ku5BjwiK5_ExNNrf8v?usp=sharing (accessed on 19 December 2025).

## Supplementary Materials

The source code for this article is available at https://github.com/cafe1930/MultispectralSegmentation (accessed on 19 December 2025).

## Abbreviations

The following abbreviations are used in this manuscript:

OSM	OpenStreetMap
L2A	Level-2A (Sentinel-2 atmospheric correction level)
RGB	Red, Green, Blue (spectral bands)
NIR	Near-Infrared
m/pix	Meters per pixel
DL	Deep Learning
CNN	Convolutional Neural Network
ViT	Vision Transformer
MAxViT	Multi-Axis Vision Transformer
DNN	Deep Neural Network
SE	Squeeze-and-Excitation (module)
NAS	Neural Architecture Search
SMP	Segmentation Models Pytorch (library)
EfficientNetB2	EfficientNet Backbone, version B2
CSPNet	Cross-Stage Partial Network
CSPDarkNet53	CSP-based DarkNet-53 backbone
VGGNet-16	Visual Geometry Group Network, 16 layers
UNetFormer	UNet-like Transformer model
U-TAE	Time-Attention Encoder (temporal segmentation model)
Sen4x	Sentinel-2 Super-Resolution Model
Swin2SR	SwinV2 Transformer for Super-Resolution
MSNet	Multispectral Semantic Segmentation Network
PSNet	Multispectral Universal Segmentation Network
SGDR	Stochastic Gradient Descent with Warm Restarts
Adam	Adaptive Moment Estimation (optimizer)
IoU	Intersection over Union
CE	Cross-Entropy (loss)
FN	False Negative
FP	False Positive
TP	True Positive
FLAIR	French Land-cover from Aerial Imagery Repository
PEER	Pacific Earthquake Engineering Research Center
QGIS	Quantum Geographic Information System
GIS	Geographic Information System
A.	Applicable
N.A.	Non-Applicable
